# RESPIRATORY MUSCLE STRENGTH IN IDIOPATHIC SCOLIOSIS AFTER TRAINING PROGRAM

**DOI:** 10.1590/1413-785220162406120752

**Published:** 2016

**Authors:** Vera Lúcia dos Santos Alves, Osmar Avanzi

**Affiliations:** 1. Universidade Mogi das Cruzes, Faculdade de Ciências Médicas, Santa Casa de Misericórdia de São Paulo, São Paulo, SP, Brazil.; 2. Faculdade de Ciências Médicas, Santa Casa de Misericórdia de São Paulo, Department of Orthopedics and Traumatology, Santa Casa de São Paulo, São Paulo, SP, Brazil.

**Keywords:** SCOLIOSIS, MUSCLE STRENGTH, EXERCISE THERAPY

## Abstract

**Objective:**

To analyze the impact of a physiotherapy protocol in maximum inspiratory and expiratory pressure in patients with adolescent idiopathic scoliosis (AIS) by manovacuometry. AIS may change the respiratory dynamics and the performance of inspiratory and expiratory muscles, affecting ventilatory capacity.

**Methods:**

Patients with AIS aged 10 to 20 years old were randomly assigned to receive an aerobic exercise-training program or no treatment. They were evaluated for respiratory muscle strength before and after the treatment period by means of manovacuometry, thorax and spine radiographs. Physical therapy exercising protocol comprised three weekly sessions including stretching and aerobic exercises during four months.

**Results:**

Forty five patients received physical therapy and 45 patients received no treatment (control group). The mean maximum inspiratory pressure (Pi_max_) was -52.13 cm H_2_0 and the maximum expiratory pressure (Pe_max_) was 62.38 cm H_2_0. There was a significant increase of Pi_max_ and Pe_max_ (p=0,000) in the group receiving physical therapy. There were no drop-outs and no adverse events in this study. Respiratory muscle strength, scoliosis and kyphosis degrees were not statistically correlated.

**Conclusion:**

Exercising is beneficial to patients with AIS, who have shown significant increases in respiratory muscle strength after physical therapy. There was no correlation between respiratory pressure and spine deformity. Level of Evidence I, High quality randomized trial.

## INTRODUCTION

The airflow limitation during physical activities is reported in patients with adolescent idiopathic scoliosis (AIS) with moderate or severe spinal curvature (> 60°).^1^ However, even adolescents with smaller curvatures and who do not exhibit restrictive respiratory pattern when compared to individuals without vertebral deformities, measured by spirometry, have less ability to perform physical activities.^2^

In the biomechanical analysis of the interaction between the spine, sternum and ribs, there is evidence that the vertebral rotation causes change in anteroposterior and transverse diameter of the chest during inspiration.^3^ ^,^ ^4^ Since respiratory movements are given by the interaction of three muscle groups, the change in the expansion capacity results in loss, because it interferes in the dynamic compliance of the thoracic complex.^5^

One way of analyzing respiratory muscle efficiency is by measuring the maximal respiratory pressures, namely the maximal inspiratory pressure (Pi_max_) which indicates the strength of the inspiratory muscles and maximal expiratory pressure, (Pe_max_), which indicates the strength of the expiratory muscles.^6^

For the purpose of standardization, a consensus for conducting respiratory muscles strength tests was established in 2002,^7^ recommending the manovacuometry test.^8^ Due to the possibility that measuring the maximum pressures may combine the muscular action of the rib cage and its elastic recoil,^6^ the evaluation is essential to patients with AIS, as these can present a mechanical disadvantage by the distortion imposed on the rib cage, which in turn is consequent to the spinal curvature.

The question to be verified was whether the modification of the respiratory dynamics alters the inspiratory performance, generating ventilatory changes as compared to normal pattern, regarding metabolic needs. During exercise,^9^ ^,^ ^10^ for example, the lower muscular strength found in patients with AIS, which typically do not have adequate physical fitness, is associated to less periphery muscle mass.^11^

Following the hypothesis that deconditioning could be the factor responsible for the muscle strength change of these patients, cardiorespiratory and musculoskeletal fitness provided by standard physical activities can benefit them ^12^ it was verified by our team, ^13^ which found improvement of respiratory function in patients with AIS submitted to aerobic training program.

The lack of studies in the literature on the behavior of the change in respiratory strength in patients with AIS and the benefit of an aerobic exercise program applied to these patients is the purpose of evaluating the impact of a physical therapy protocol on maximal expiratory and inspiratory strength applied to patients with AIS assessed by manovacuometry.

## MATERIALS AND METHODS

This is a prospective randomized study which evaluated patients with AIS and curvature ≥ 45°, with surgical indication, consecutively diagnosed at the Departments of Orthopaedics and Physical Therapy of a public university hospital from January 2008 to February 2009.

Patients were randomly divided into two groups: group I, the control group, consisting of patients with AIS, and group II of patients with AIS who underwent a physical therapy protocol. All patients signed an Informed Consent Form and the study was approved by the Research Ethics Committee under number 301/08.

Patients with AIS with curvature ≥ 45 °, aged 10 to 20 years old, candidates for surgical correction of spinal deformity were included at the study that evaluated spinal deformity, as well as the angle of kyphosis by anteroposterior and profile radiographs.^14^

We excluded patients who had undergone previous surgery for spinal deformity correction, those with cognitive and musculoskeletal changes that might interfere in comprehension and performance of tests and those who claimed to perform standardized physical activity for more than 30 minutes three times a week. Patients were divided into groups by random selection of opaque envelopes sealed and numbered sequentially.

### Evaluation of respiratory muscle strength

One day before and one day after the period of application of the exercise protocol, all patients were evaluated by using the manovacuometer (*Comercial Médica* ^(r)^ to measure Pi_max_ and Pe_max_. Following consensus guidance of the respiratory muscle testing,^7^ pressure measurements were performed with the patient seated, with the chest and feet flat, using a nose clip. The patient was instructed to hold the manovacuometer and tighten the mouthpiece firmly against the lips, preventing air leakage, making a maximum inspiration from the residual volume to measure Pi_max_, and a maximum expiration from total lung capacity to determine Pe_max_.^7^

In each evaluation, were performed three measures of Pi_max_ and Pe_max_, under the supervision and direction of a physiotherapist with resting intervals of 30-60 sec between measurements. We considered the highest value recorded.

### Physical therapy protocol

Patients in group II underwent a physical therapy protocol during four months, based on the proposals by Bouchard and Shepard^15^ and Covey et al, ^16^ with three weekly sessions with 60 min intervals followed by the physiotherapist and divided into three steps:

• 10 min of warming up (low intensity stretching and aerobic exercises such as slow and progressive walking);
• 40 min of aerobic exercise on the treadmill or exercise bike, and training intensity was maintained at 60-80% of the maximum heart rate;
• 10 min cool-down and relaxation (stretching and aerobic exercises with low energy expenditure and relaxation techniques).

Each patient of group II was, therefore, submitted to 48 exercise sessions performed at the same physical therapy clinic, using deletar of the same equipment in all sessions and patients.

The control group patients were not subjected to any kind of physical exercise. They were instructed to perform their daily activities normally, and were submitted to a new assessment four months after the initial evaluation.

The number of patients in each treatment group followed the sample size calculation. Statistical analysis was performed using SPSS (Statistical Package for Social Sciences), version 13.1. Paired t-test and variance homogeneity were performed to observe the strength of both groups and the Kolmogorov-Smirnov normality test was applied to establish the correlation between the scoliosis angle, kyphosis and respiratory pressures. Statistical significance was established as p<0.05.

## RESULTS

During the study period, 90 patients were included, 45 in the control group (I) and 45 in the study group (II). Table 1 shows the result of the evaluation of patients in both groups. There was no significant difference between the two groups regarding age and spine angle measurement at the time of inclusion in the study, as well as the comparison between Pi_max_ and Pe_max_ (baseline). 

**Table 1:** Mean and standard deviation of age and evolution of spine deformity in all patients before intervention.

	Age (years old)	Scoliosis angle	Kyphosis angle
Group I (control group)	14.27±2.02	60.62°±16.03° (range, 45°-138°)	35.02°±13.63^o^ (range, 12°-69°)
Group II (study group)	14.34±1.95	57.64 ± 12.23° (range, 45°-110°)	31.29°±12.23^o^ (range, 9°-68°)

Mean Pi_max_ at start of all patients was -36.04±7.11cm H_2_O and the mean Pe_max_ was 43.91±4.53cm H_2_O, ranging, respectively, between -15 and -50 and between 31 and 51. 

**Figure 1:**
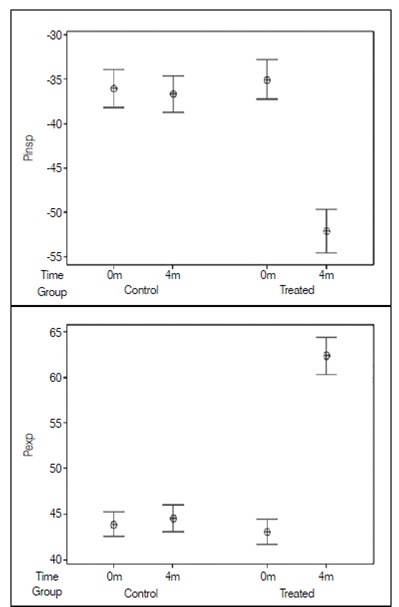
Means and confidence intervals of maximal inspiratory and expiratory pressure in patients with adolescent idiopathic scoliosis.

Every patient in group II completed all planned physical therapy sessions. The measurement of respiratory pressures before the physiotherapy protocol in group II showed mean Pi_max_ -35.04±7.39 cm H2O and mean Pe_max_ 43.11±4.65 cm H2O, ranging from - 15 and -47, and 32 and 51, respectively. After completion of the physical therapy protocol, a new assessment was performed and showed a significant increase in the values of Pi_max_ and Pe_max_ in group II, with p = 0.000 for both variables, mean Pi_max_ was -52.13±8.33 cm H2O (SD =) and Pe_max_ was 62.38±6.74 cm H2O. (Figure 1) There was no difference between the mean values of patients in group I in the first and in the last assessment.

**Figure 2:**
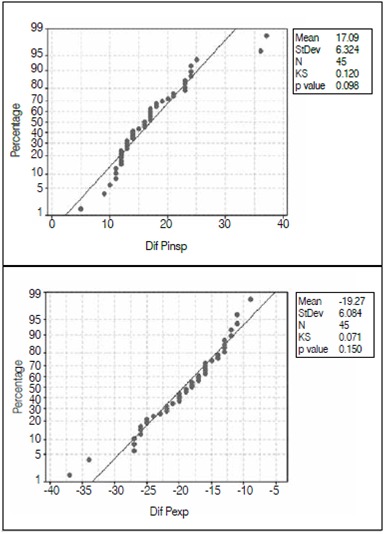
Dispersion for correlations between Pi_max_, Pe_max_, scoliosis and kyphosis.

Correlations between respiratory muscle strength, scoliosis and kyphosis were weak in all groups and time points studied; there was no significant association in any analysis in group II (Table 2) and there were no linear relationships between the variables studied. (Figure 2)

**Table 2:** Mean values of comparison of maximal inspiratory pressure (Pi_max_) e maximal expiratory pressure (Pe_max_) at the initial stage and after four months of physical therapy protocol in patients with adolescent idiopathic scoliosis.

Measurements	Scoliosis	Kyphosis
Pi_max_ - initial	0.259	0.108
*p*	0.086	0.481
Pe_max_ - initial	-0.149	-0.183
*p*	0.329	0.230
Pi_max_ - 4 months later	0.276	-0.129
*p*	0.067	0.400
Pe_max_ - 4 months later	-0.114	0.260
*p*	0.456	0.085

Pe_max_: maximal expiratory pressure; Pi_max_: maximal inspiratory pressure.

## DISCUSSION

The assessment of lung function by spirometry not always characterizes restrictive lung disease in AIS.^10^ Therefore, the literature searches other changes to explain the lower functional capacity in these patients.^1^

The ventilatory performance during physical activities depends not only on the lungs' mechanical properties, but also the interaction between complacency, elastance and muscle action.^6^ The relationship between pressure and strength is complex because the rib geometry is responsible for the conversion of strength into pressure, and this is directly dependent on the mechanical characteristics of the chest, abdominal compliance and interaction of respiratory muscles.^7^

Ninety adolescents with AIS were compared to 40 adolescents without any spinal deformity in a study by Alves dos Santos et al.,^13^ who observed a weak correlation between the scoliosis angle and muscle strength presented by the AIS group. These findings are corroborated by the present work, in which the strength measurements are not correlated with the angular value of the spinal deformity. This suggests that respiratory strength in patients with AIS may be reduced by physical deconditioning.^1^ ^,^ ^2^

This possibility was already suggested by Kearon et al.,^11^ who observed that individuals with scoliosis showed reduced performance in aerobic exercises, with different physiological responses in varying degrees of deformity and lower muscle mass as compared to patients with AIS and individuals without spinal deformity undergoing incremental test on a cycle ergometer.

For patients with chronic lung disease, the effectiveness of physical therapy reabilitation in systemic manifestations has already been defined, including depletion of skeletal muscle mass.^17^ Thus, the implementation of rehabilitation protocols with aerobic exercises for patients with AIS should be encouraged, according to positive data found by many authors^1^ ^,^ ^13^ ^,^ ^18^ and endorsed by this study, which observed increased respiratory muscle strength after the training program.

The account of lower peripheral muscle mass found in AIS is important because the level of body mass has a clear relationship to lean body mass and, therefore, with the metabolically active tissue capable of producing work. Similarly, the increase of inspiratory muscle strength is probably related to a higher residual ability to support metabolic and ventilatory demands of physical training.^18^

In 2003, Zaba^18^ studied respiratory function and limitation to perform exercise in 70 patients with AIS, as compared to 22 adolescents without spinal changes, and he did not observe any significant increase of lung volume in both groups after the completion of a rehabilitation program. In AIS patients, he found increased voluntary ventilation, given the improvement in respiratory muscle strength, which was also found in our study

It is known that aerobic exercise in patients with chronic obstructive pulmonary disease increases the concentration of oxidative mitochondrial enzymes, the capillarity of the trained muscles, the aerobic threshold, and maximum VO_2_, reducing the recovery time of creatine phosphate. Therefore, it allows better capacity in carrying out the exercise,^19^ demonstrating that the aerobic activity in these patients is more effective than the specific training of the respiratory muscles.^9^

According to Smyth et al.,^19^ when training provides control of breathing pattern, increased strength and inspiratory muscle endurance can translate into clinical improvement of the respiratory system functional status of patients with chronic lung disease, as it was observed in this study with AIS patients.

The increase of the maximum respiratory muscle strength can be explained according to Helbling et al., ^20^ by the cardiorespiratory adaptation to training intensity, which promotes the recruitment and better function of oxidative muscle fibers, unlike the statement by Lacasse et al., ^21^ who claimed that physical activity improves peripheral and respiratory muscles without specific training of the muscles, although the literature is emphatic on the need for standardizing physical training.^15^ ^-^ ^17^

## CONCLUSION

The therapy protocol benefited patients with AIS, which had a significant improvement of their respiratory muscle strength, as compared to patients with AIS who did not undergo the exercise protocol.
